# *Enterococcus faecalis* Endogenous Endophthalmitis from Valvular Endocarditis

**DOI:** 10.1155/2013/174869

**Published:** 2013-07-01

**Authors:** Sidnei Barge, Renata Rothwell, Rosário Varandas, Luís Agrelos

**Affiliations:** Department of Ophthalmology, Centro Hospitalar Vila Nova Gaia/Espinho, Rua Conceição Fernandes, 4434-502 Vila Nova Gaia, Portugal

## Abstract

We report a case of a 74-year-old female, with a mitral heart valve, who presented with pain and blurred vision in the right eye for 2 days. Her visual acuity was light perception (LP) in the right eye and 20/40 in the left eye. Slit lamp examination showed corneal edema and hypopyon, and a view of the right fundus was impossible. Echography showed vitreous condensation. One day after presentation, the patient developed acute lung edema requiring hospitalization, so she was not submitted to vitreous tap and intravitreal treatment. The cardiac and systemic evaluations revealed a mitral endocarditis secondary to *Enterococcus faecalis*. The patient improved systemically with treatment with gentamicin, vancomycin, and linezolid. Her visual acuity remained as no LP, and her intraocular pressure (IOP) has been controlled with brimonidine bid despite developing a total cataract with 360° posterior synechia. A cardiac source for endogenous endophthalmitis should be considered in the presence of a prosthetic cardiac valve. The treatment and followup must be made in cooperation with a cardiologist specialist, but the ophthalmologist can play a key role in the diagnosis.

## 1. Introduction

Since the advent of antibiotics, endogenous bacterial endophthalmitis (EBE) is a rare but visually devastating disease [1–3]. Most publications on this subject have been limited to small case series or reports, underscoring the infrequent occurrence of this condition [2].

It arises as a result of hematogenous spread from a septic focus distant to the eye [1]. 

EBE is associated with underlying medical conditions such as diabetes, cardiac disease, and malignancy in up to 90% of patients [4].

We present a case report at the Vila Nova Gaia Hospital and review the literature.

## 2. Case Report

**Figure 1:**
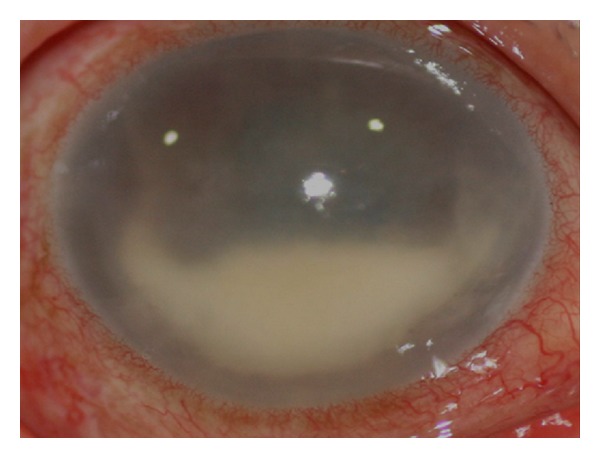
Corneal edema and a white hypopyon filling one-third of the anterior chamber.

A 74-year-old female reported pain and blurred vision in the right eye for 2 days. She had dyspnea, orthopnea, weight loss, and malaise during the previous month. There was no ocular history, but three years before, she had been submitted to a mitral valve replacement with a mechanical valve. At presentation, her visual acuities were 20/25 in the left eye and light perception (LP) in the right eye. The left eye was normal. A relative afferent pupillary defect was present in the right eye, and this eye was hypotonous. There was corneal edema, fibrin in the anterior chamber, and a white hypopyon filling one-third of the anterior chamber (Figure 1).

**Figure 2:**
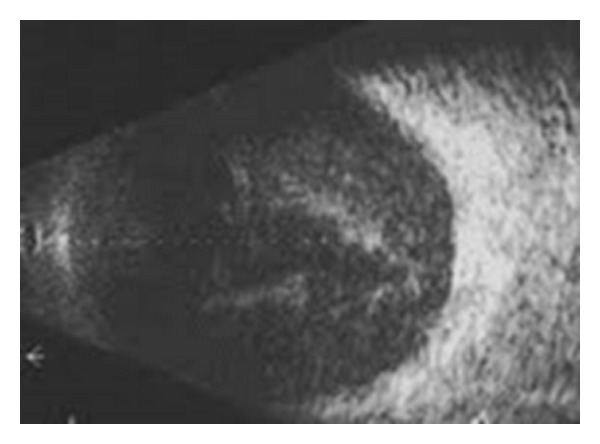
Ultrasonography OD—diffuse vitreous condensation.

The fundus was not visible, but ultrasonography of the posterior segment revealed diffuse vitreous condensation (Figure 2).

**Figure 3:**
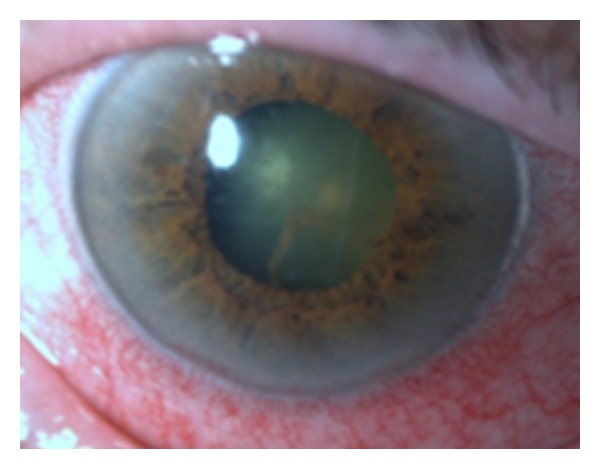
Biomicroscopy OD after 2 weeks—cataract and no corneal edema or hypopyon.

With a diagnosis of endophthalmitis, the patient required hospitalization with topical and intravenous vancomycin and ceftazidime, topical atropine, and oral prednisolone (1 mg/kg/day). A vitreous tap and intravitreal vancomycin (1 mg/0.1 mL) and ceftazidime (2 mg/0.1 mL) were planned for the next day. However, the patient developed acute lung edema with atrial fibrillation requiring hospitalization in the intensive care unit. The blood culture showed *Enterococcus faecalis* sensitive to gentamicin and vancomycin. After five days, the patient experienced spacial and temporal disorientation with aggressive behavior, and she suspended topical atropine with neurological improvement. After two weeks, she had no LP in the right eye and ocular pressure was impossible to measure due to pain. The biomicroscopy showed cataract and no corneal edema or hypopion (Figure 3).

The fundus was not visible. The physical examination of the left eye was unremarkable.

**Figure 4:**
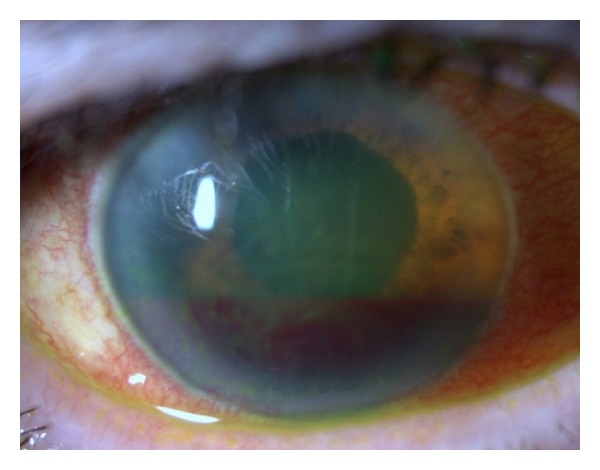
Biomicroscopy OD after 5 weeks—Descemet's folds and hyphema without hypopyon.

Due to her past medical history and after medical stabilization, transesophageal echocardiography was performed, showing cardiac mitral atherosclerotic valve vegetation. We made a diagnosis of endogenous endophthalmitis secondary to prosthetic endocarditis. She was hospitalized with intravenous vancomycin and gentamycin for 3 weeks and oral linezolid at home for 2 months. After five weeks, the patient complained of right ocular pain. At examination, ocular pressure was 46 mmHg and biomicroscopy revealed Descemet's folds and hyphema without hypopyon (Figure 4).

The patient started oral acetazolamide (250 mg/day) and brimonidine bid that controlled IOP.

**Figure 5:**
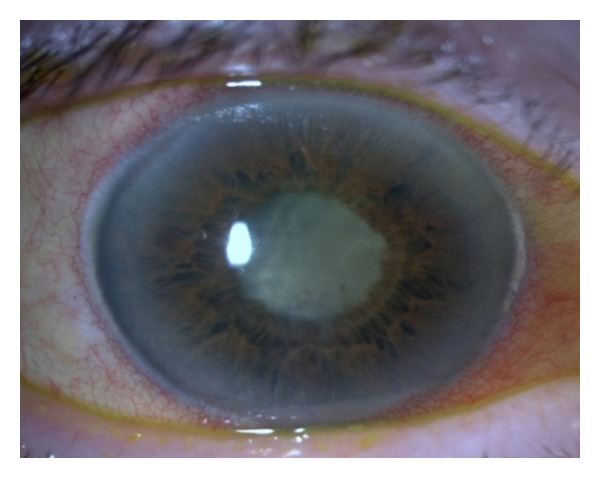
Biomicroscopy OD—total cataract and posterior synechia 360°.

The patient recovered systemically with resolution of the infective endocarditis. During the one-year followup, the patient has been clinically stable, with no ocular pain. Her visual acuity remained as no LP and her IOP has been controlled (20 mmHg) with brimonidine bid despite developing a total cataract with 360° posterior synechia. (Figure 5).

## 3. Discussion

Enterococci are the leading cause of subacute endocarditis [5]. *Enterococcus faecalis* is a gram-positive streptococci, a natural inhabitant of the mammalian gastrointestinal tract and is found in soil, sewage, water, and food frequently through faecal contamination. It is an opportunistic pathogen which is a major cause of urinary tract infections, bacteremia, and infective endocarditis [6].

Possible reasons for the apparent predisposition to infection of the retinal circulation include the fact that it is an end-artery system, the avascular vitreous may act as a bacterial reservoir, and the blood-ocular barrier may interfere with the host inflammatory response. It is not known why some patients develop EBE but it may relate to the size of the inoculum, immunodeficiency, the virulence of the organism, and comorbidity [7].

The right eye was twice as likely to be affected as the left eye. It was postulated that this is because of more proximal and direct arterial blood flow to the right carotid [8].

In our case, endophthalmitis was the first manifestation of a systemic disease. Okada and colleagues reported that half of patients had no systemic symptoms, and over half saw an ophthalmologist first [4].

Unfortunately, in our case the patient has no light perception. *Enterococcus faecalis* is known to be a virulent pathogen associated with endophthalmitis. Extracellular toxins, such as cytolysin, and superoxide production are known to be contributing factors to the pathogenic potential of *E. faecalis* [9]. Furthermore, possession of variable traits, such as plasmid encoded cytolysin, may provide a colonization advantage for *E. faecalis* [10]. The visual prognosis of *E. faecalis* endophthalmitis is generally very poor, with almost 50% of final visual outcomes being LP to no LP and another 35%, 5/200 to hand movements [11]. Enucleation occurs in approximately 50% to 90% of cases [6].

In this case, it was not possible to administer intravitreal antibiotics and perform vitreous tap due to severe cardiac decompensation. However, blood culture showed *Enterococcus faecalis*. The use of intravitreal and topical antibiotics is controversial. Although some advocate the delivery of the antibiotic into the vitreous cavity to destroy the causative microbe directly and rapidly during a vitreous tap for culture, others have countered that, unlike postoperative bacterial endophthalmitis, the main source of infection is not the eye, but of a hematogeneous origin. Thus, if the bacteria are capable of penetrating the blood-ocular barrier to infect the eye, the antibiotics should enter the eye effectively to sterilize it [1]. Blood culture is the most reliable way of establishing the diagnosis. In four large series of EBE, blood cultures were more likely to be positive than vitreous [4, 12–14].

Unlike that in acute postoperative endophthalmitis [15], it is important to administer intravenous antibiotics in addition to intravitreal antibiotic injections in the management of endogenous endophthalmitis because parenteral antibiotics are often required to treat the primary infective focus and the concurrent septicemia [2].

Our patient had a prosthetic mitral valve for three years and significant atherosclerotic changes in her echocardiography. Endocarditis often affects degenerative valves and can occur in 33.3% of patients with endophthalmitis [16–18]. It is likely that these degenerative valves, in elderly patients, have atherosclerotic changes that may predispose patients to bacterial adherence and proliferation [1].

For the treatment of endocarditis, our patient received intravenous combination therapy with vancomycin and gentamycin for 3 weeks and then oral linezolid for 2 months. Because of the difficulty in delivering high concentrations of antibiotics to the heart valves in endocarditis, multiple-drug therapy is a recommended treatment strategy [19]. In one study on the development of synergy with multiple systemic drugs, the greater inhibitory or bactericidal activity of multiple drugs in combination was greater than would be expected from the sum of each drug alone. [20, 21]. Increasing resistance to currently available antibiotics continues to be a serious problem in the treatment of *E. faecalis* infections. Commonly used in the empiric treatment of acute endophthalmitis [22], vancomycin generally is effective against gram-positive organisms, including *E. faecalis* [15], but vancomycin-resistant enterococci have been reported [23–25].

In conclusion, endophthalmitis caused by *Enterococcus faecalis* is an infrequent and unusually virulent infection with a typically poor prognosis even with aggressive treatment.

As in a significant percentage of patients the first manifestations of endogenous endophthalmitis are ocular symptoms, the ophthalmologist can play a key role in diagnosis. In a patient with a prosthetic cardiac valve, an endocarditis should be suspected as source of an endogenous endophthalmitis.

When there is a cardiac source, a multidisciplinary team including cardiologist should be involved in treatment and followup.
